# *Ex vivo* Induction of Apoptotic Mesenchymal Stem Cell by High Hydrostatic Pressure

**DOI:** 10.1007/s12015-020-10071-0

**Published:** 2020-10-31

**Authors:** Tien Minh Le, Naoki Morimoto, Nhung Thi My Ly, Toshihito Mitsui, Sharon Claudia Notodihardjo, Shuichi Ogino, Jun Arata, Natsuko Kakudo, Kenji Kusumoto

**Affiliations:** 1 Department of Plastic and Reconstructive Surgery, Kansai Medical University, 2-5-1 Shin-machi, Hirakata, Osaka 573-1010 Japan; 2 Department of Orthopaedics, SAIGON International Trauma Orthopaedics (SAIGON - ITO) Hospital, 140C Nguyen Trong Tuyen, Phu Nhuan District, Ho Chi Minh City, 72217 Vietnam; 3 Department of Plastic and Reconstructive Surgery, Graduate School of Medicine, Kyoto University, 54 Kawaharacho, Shogoin, Sakyo-ku, Kyoto, 606-8507 Japan; 4 Department of Dermatology, Kansai Medical University, 2-5-1 Shin-machi, 573-1010 Hirakata, Osaka Japan

**Keywords:** Apoptosis, Extracellular vesicles, Mesenchymal stem cell (MSC), High hydrostatic pressure (HHP), Tissue engineering

## Abstract

Among promising solutions for tissue repair and wound healing, mesenchymal stem (or stromal) cells (MSCs) have been a focus of attention and have become the most clinically studied experimental cell therapy. Recent studies reported the importance of apoptosis in MSC-mediated immunomodulation, in which apoptotic MSCs (apoMSCs) were shown to be superior to living MSCs. Nowadays, high hydrostatic pressure (HHP), a physical technique that uses only fluid pressure, has been developed and applied in various bioscience fields, including biotechnology, biomaterials, and regenerative medicine, as its safe and simply operation. In the current study, we investigated the impact of HHP treatment on human bone marrow-MSC survival and proliferation. Based on the detection of executioner caspase activation, phosphatidylserine exposure, DNA fragmentation (TUNEL) and irrefutable ultrastructural morphological changes on transmission electron microscopy (TEM), our data revealed that HHP treatment induced complete apoptosis in MSCs. Notably, this technique might provide manipulated products for use in cell-based therapies as manufacturing capability expands. We hope that our findings will contribute to the improvement of MSCs or EVs in translational research development.

**Figure:**
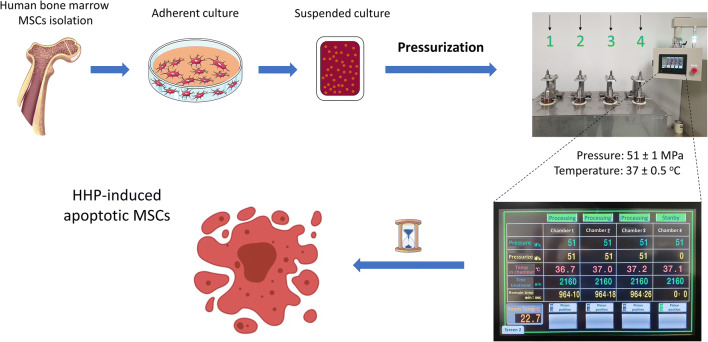
Graphical Abstract

Graphical Abstract

## Introduction

Mesenchymal stem/stroma cells (MSCs, a term first coined by Caplan) are nonhematopoietic stem cells derived from bone marrow [1] that have multipotent differentiation capacity to mesodermal tissues, such as bone, tendon, cartilage, muscle, and fat [2]. This finding was expected to open the era of replacing or repairing damaged tissues of mesenchymal origin. Since then, MSCs have been a focus of attention for their broad-ranging clinical potential, particularly in the rapidly growing field of regenerative medicine. Over the past decade, numerous advances have been made in the development of MSCs as a therapy for a highly diverse group of diseases, including cardiac, neural, and orthopedic diseases. However, the treatment effect of living MSCs intravenous therapy only found in 40–50% of treated patients [3]. There is evidence to explain above result that after intravenous infusion, the majority of MSCs are trapped in the microvascular network of the lungs and tissues (due to their large size), [4] where they are then quickly phagocytosed by blood-derived monocytes and neutrophils [5]. Recently, Galleu et al. reported that infused living MSCs are subject to perforin-induced apoptosis through recipient cytotoxic cells [6]. As cytotoxic cell-induced apoMSCs were reported to be essential for MSC-mediated immunomodulation, the allogenic components might be of secondary importance. These findings emphasize the importance of apoptosis in MSC-mediated immunomodulation and might explain previous study results, in which apoptotic MSCs (apoMSCs) were shown to be superior to living MSCs [7]. Furthermore, previous studies have suggested that viable MSCs could favor tumor growth *in vivo* by involving the formation of carcinoma-associated fibroblasts (CAFs) [8, 9]. As dead MSCs have no active cell metabolism, it can be assumed that they do not differentiate into CAF-like cells with the corresponding secretion of growth factors, cytokines, and chemokines [5, 7]. Accordingly, it is considered that dead or apoptotic MSCs may be important materials for new approaches in the development of cell-based therapies [6, 10, 11]. The development of such therapies also requires a new method for effectively inducing apoptotic MSCs that can be applied in large-scale manufacturing.

High hydrostatic pressure (HHP) is a physical technique that uses only fluid pressure to inactivate cells or tissues, without the use of any chemical reagents [12, 13]. Due to the safety and simplicity of operation, HHP has been developed and applied in a various bioscience fields, including biotechnology, biomaterials, tissue engineering and regenerative medicine [14, 15]. While development HHP treatment techniques, we succeeded in inducing complete apoptosis of human dermal fibroblasts and skin tissue by exposure to persistent HHP at 50 MPa for 36 hours [16]. Then, in *in vivo* transplantation, the apoptotic skin showed superior wound healing and regeneration in comparison to the conventional method. We considered that the method might be effective for the induction of apoptosis of MSCs *ex vivo* and that it may contribute to the progression of apoMSC studies in translational research. Thus, in this study, we explored the possibility of using hydrostatic pressure to induce apoptosis of human bone marrow-derived MSCs.

## Materials and Methods

### The Isolation and Culture of Bone Marrow MSCs

**Fig. 1:**
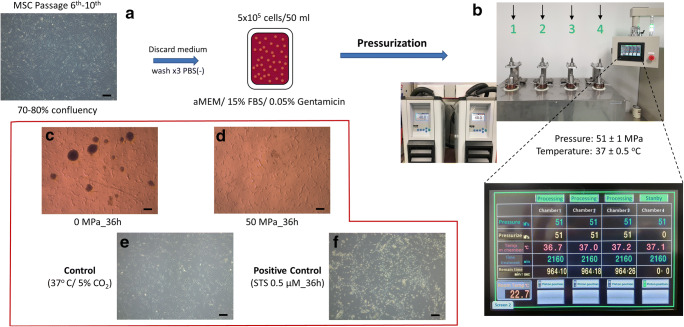
Schematic description of the pressurization processing. Adherent MSCs after several passages (**a**) in an incubator were trypsinized and transited to suspend culture in a sealed plastic bag. Each bag was transferred to an isostatic chamber (No. 1–4) filled with warm tap water then pressurized using an automated HHP device. (**b**) The enlarged screen monitor shows the input setting and status of the environmental condition inside each chamber. The timer countdown started after pressure setting was reached. The system automatically reduces pressure to 0 when finished. (**c**) Immediately after pressurization, a large number of viable MSC clusters were found in the 0 MPa_36 h group, but only cell debris was observed in the 50 MP_36 h group. (**d**) Micrographs show the difference between MSCs in the incubator (control, **e**) and MSCs treated with 0.5 µM STS for 36 h (positive control, **f**). All the images were made by authors and had permission for using or publishing

MSCs were derived from human bone marrow and kindly supplied by Japan Tissue Engineering company (Lot No. BKXH-1-P5-79,80; J-TEC, Japan Tissue Engineering Co., Ltd., Gamagori, Aichi, Japan). After rapid thawing in a water bath at 37 °C, 2 × 10^6^ cells per cryovial were seeded and cultured on a 4 × 10-cm dish (Falcon; Corning Inc., Corning, NY, USA) with BMP01(-) culture medium consisting of aMEM, 15% FBS, and 0.05% Gentamicin (Lot No. 190,306; J-TEC Co., Ltd.). These cells were then incubated at 37 °C in a normoxic incubator under 5% CO_2_, and presented fibroblast-like morphology, as shown in Fig. 1a. The medium was changed every 2–3 days and MSCs were subcultured when they reached 80–90% confluence. MSCs at passages 6–10 were used in the experiment.

### Inducing Apoptosis of MSCs and Collection Technique

When the culture reached 70%-80% confluence, adherent cells were washed 3 times with phosphate-buffered saline without calcium and magnesium (PBS; Takara Bio Inc., Kusatsu, Japan) and then dissociated using TrypLE Express (Gibco, Thermo Fisher Scientific Inc.). As shown in Fig. 1, a total of 5 × 10^5^ cells in suspension were then dissolved in a sterile plastic bag filled with 50 ml of culture medium, which was subsequently sealed. These bags were then subjected to pressurization for 36 h using an automated custom-made HHP device. In brief, when a stable temperature of 36–37 °C was reached in all isostatic chambers, a sealed bag was placed into each chamber, which was then filled with warm tap water and closed tightly. The pressure was then increased to 51 MPa in chambers 1-2-3, while no pressure was applied in chamber 4 (0 MPa, Fig. 1b). After the specific pressure magnitude was reached, a countdown on screen was started until 0, at which point the pressure was automatically reduced by the system. The remaining MSCs were parallel cultured in a 10-cm culture dish in an incubator at 37 °C, for 36 h, under 5% CO_2_, as a control (Fig. 1e), while MSCs treated with 0.5 µM Staurosporine (STS; #ab120056; Abcam Co. Ltd., MA, USA) for 36 h were used as a reference (Fig. 1f) [17]. After pressurization, all of the medium (including cells) in each bag was aspirated and then transferred to 50-ml conical tubes (Falcon, Corning Inc.). After centrifugation at 1500 *g* for 3 min, the supernatant was discarded, then cell pellets were washed with PBS and resuspended in 1 ml of fresh medium for downstream investigation. As we detected the appearance of MSC clusters in the 0 MPa_36 h group (Fig. 1c), the cell pellets were washed twice with PBS and then detached by 5 min of exposure to 0.5 ml of TrypLE Express in the incubator. In the STS group, as we found no adherent cells in the 10-cm culture dish after 36 h of treatment, the supernatant was aspirated then transferred to 15-ml conical tubes (Falcon, Corning Inc.) and centrifuged for the collection of apoMSCs.

### The Flow Cytometry Of apoMSCs Induced by HHP

Briefly, 2–3 × 10^5^ cells per sample were collected and washed 2 times with PBS. For the detection of PtdSer membrane translocation, MSCs were then resuspended in a mixture of 200 µl of Assay Buffer, 2 µl of Apopxin Green Indicator, 1 µl of 7-AAD and 1 µl of CytoCalcein 450 (Apoptosis/Necrosis Detection Kit; #ab176749; Abcam Co., Ltd.) as manual instructions. The samples were then kept protected from light at room temperature (RT) for 30–60 min, and 2 × 10^4^ cells were subsequently archived for flow cytometry on a BD FACSCanto II (BD Biosciences, San Jose, CA, USA) using the Flowjo software program (Flowjo LLC, BD Biosciences). The amounts of early apoMSCs (Apopxin^+^/7-AAD^−^) were analyzed and compared.

In addition, after HHP treatment, 1 ml of medium containing approximately 2–3 × 10^5^ cells per sample was mixed with 1 µl of CellEvent Caspase-3/7 Green Detection Reagent (CellEvent Caspase-3/7 Green Flow Cytometry Assay Kit; Thermo Fisher Scientific, Inc.). The mixture was then vortexed and incubated for 60 min at RT, protected from light. In the final step, 1 µl of 1 mM SYTOX AADvanced dead cell stain solution (CellEvent Caspase-3/7 Green Flow Cytometry Assay Kit; Thermo Fisher Scientific Inc.) was added, and then 2 × 10^4^ cells were archived for analysis on a BD FACSCanto II (BD Biosciences) using the Flowjo software program (Flowjo LLC, BD Biosciences).

### Immunofluorescence Microscopy

After pressurization, 1 × 10^5^ cells per sample were washed with PBS, resuspended in fresh medium, and seeded in a 35-mm glass-bottom dish (Matsunami Glass Ind., Ltd., Osaka, Japan), after which they were incubated for 3 hours at 37 °C under 5% CO_2_ to encourage attachment to the dish bottom. For the observation of apoptosis/necrosis, the medium was gently discarded, and cells were washed 2 times with 200 µl of Assay Buffer. The cells attached to each glass-bottom dish were then incubated with a mixture of 200 µl of Assay Buffer, 2 µl of Apopxin Green Indicator, 1 µl of 7-AAD and 1 µl of CytoCalcein 450 (#ab176749; Abcam) at RT for 30–60 min. Finally, images were acquired using a confocal laser scanning microscope by the combination of Violet, FITC and Alexa Fluor 647 signals (Fluoview FV3000; Olympus Co., Tokyo, Japan).

To measure DNA fragmentation, we used the DeadEn Fluorometric TUNEL System (#G3250; Promega Corp., WI, USA) according to the instruction manual. In brief, 2–3 × 10^5^ cells per sample were resuspended in fresh medium, then cultured in a 35-mm glass-bottom dish. After incubation for 30–60 min to encourage dish attachment, these cells were gently washed twice with PBS. Next, they were fixed by 4% methanol-free formaldehyde solution in PBS (pH 7.4) for 25 minutes at 4 °C, then immersed in 0.2% Triton X-100 solution for 5 minutes in order to induce cell permeabilization. Next, 200 µl of Equilibration Buffer was added to equilibrate at room temperature for 5–10 minutes. After most of the Equilibration Buffer was removed by tissue paper, 50 µL of prepared rTdT incubation buffer was added to each sample. The dish was then covered with aluminum foil to protect from light and incubated at 37 °C for 60 minutes inside a humidified chamber to allow the tailing reaction to occur. The reaction was stopped by immersing sample in 2X SSC for 15 minutes at RT, then it was washed three times with PBS to remove unincorporated fluorescein-12-dUTP. Finally, samples were counterstained with Hoestch 33,342 solution (Dojindo Molecular Technologies, Inc., Kumamoto, Japan) and visualized using FV3000 confocal laser microscope.

### Identification of Pressurized MSCs by TEM

Only the MSCs treated for 36 h at 0 and 50 MPa were observed. The specimens were prepared following the protocol described in our previous study [18]. After collection, pressurized or non-pressurized cells were fixed with fixative solution consisting of 2% glutaraldehyde, 0.1 M sodium cacodylate and 1 mM CaCl_2_ at 37 °C, pH 7.4, for 30 min. To stop fixation, they were then washed 2 times with cacodylate buffer (0.1 M sodium cacodylate and 0.2 M sucrose) at 4 °C, pH 7.4, for 10 min. Next, samples were post-fixed with 1% osmium tetroxide (OsO_4_) in cacodylate buffer at 4 °C, pH 7.4, for 30 min and dehydrated by ethanol. Then, they were infiltrated in Epon 812 resin and polymerized at 45 °C for 12 h, 55 °C for 24 h and 45 °C for 12 h. Consequently, specimens were cut into ultrathin sections and observed by TEM (JEM-1400Plus; JEOL Ltd., Tokyo, Japan).

### Viability Assessment of MSCs After Pressurization

Cell viability can be explored directly through the presence of ubiquitous intracellular esterase activity that cleave moieties from a lipid-soluble nonfluorescent probe to yield a fluorescent product [19]. CytoCalcein 450 is an optimal dye that is sequestered in the cytoplasm of cells with an intact membrane; thus, it is able to distinguish live cells from apoptotic or necrotic cells in the assessment of whole cell populations. By the combination of CytoCalcein Violet 450 staining provided in the Apoptosis/Necrosis Detection Kit (#ab176749; Abcam), we probability detected and analyzed the difference of MSC viability after treatment. Furthermore, the LIVE/DEAD Reduced Biohazard Cell Viability Kit (#L-7013, Invitrogen Co., Waltham, MA, USA), a basic two-color fluorescence assay for testing viability based on permeability, was additionally used to differentiate live and dead cells. For staining, cell pellets in each sample were immersed in a 200 µL mixture consisting of SYTO 10 Green, DEAD Red in HBSS (1:500 dilution) and incubated in complete darkness for 15 minutes at RT. Next, after centrifugation and discarding the supernatant, samples were fixed with 500 µL of 4% glutaraldehyde in HBSS for 60 minutes. Lastly, samples were centrifuged and the fixative was removed; cell pellets were resuspended in the same volume of HBSS and subjected to an analysis on a BD FACSCanto II (BD Biosciences) using the Flowjo software program (Flowjo LLC, BD Biosciences).

To assess the irreversible MSC death and proliferation, 1 × 10^4^ cells in 1 mL of culture medium were seeded into a 24-well cell culture plate (Falcon; Corning Inc.) and observed for 7 days without changing the medium; during this time the plates were incubated at 37 °C under 5% CO_2_. The morphology and proliferation of cultured MSCs were observed at specific times (3 h, 1 day, 3 days, and 7 days) by an inverted microscope (Carl Zeiss Co., Ltd, Oberkochen, Germany). At the same time, a WST-8 (Colorimetric Cell Viabilty) assay was used to quantitatively measure the proliferation of MSCs. In brief, a 100-µL aliquot of each cell suspension (1 × 10^4^ cells, n = 7 per group) was added to four 96-well plates (Falcon; Corning Inc.), then the plates were incubated at 37 °C under 5% CO_2_. At the specific evaluation time points, 10 µL of Cell Counting Kit-8 (CCK-8; Dojindo, Inc.) was added to each well and the plates were incubated at 37 °C for 2 h in accordance with the manufacturer’s instructions. Then, the plate was gently shaken, and the absorbance of medium was determined at a wavelength of 450 nm using an EnSpire Multimode Plate Reader (PerkinElmer Co., Ltd., Waltham, MA, USA). The absorbance of medium alone in the same plate-well was used as a blank (n = 7).

### Statistical Analyses

Results are shown as the mean values of at least three independent experiments and standard deviation (SD) is represented by bars. The Tukey-Kramer post hoc test was used to estimate the significance of differences among groups with the Prism software program (ver. 7.03; GraphPad Software, Inc., San Diego, CA, USA). Statistical significance was defined as *p < 0.05, **p < 0.01, ***p < 0.001, and ****p < 0.0001.

## Results

### Phosphatidylserine (PtdSer) Externalization of Pressurized MSCs

**Fig. 2:**
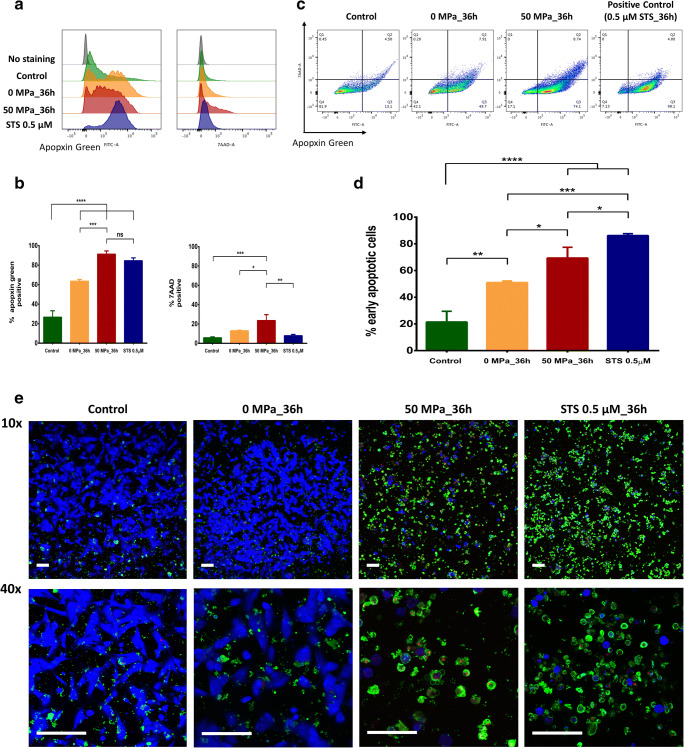
Phosphatidylserine externalization of pressurized MSCs. **a** Representative histograms of Apopxin Green staining results show the different fluorescence expression of the 0 MPa, 50 MPa and STS groups in comparison to the control group. There was no clear difference in the fluorescence of 7-AAD. **b** The comparison of the Apopxin Green-positive proportion reveals a significant increase in the treatment groups in comparison to control (*p* < 0.0001, n = 3). The FITC-positive ratio in the pressurized group was higher than that in the non-pressurized group and was no significantly different from that in the STS group. The 7-AAD positive proportion of the 50 MPa_36 h group was higher than that in other groups (*p* < 0.05, n = 3). **c** The flow cytometry diagram shows the dynamic transition of MSCs from living cells (FITC^− ve^/7-AAD^− ve^) to an apoptotic cells (FITC^+ ve^), even in the control group. In the 0 MPa_36 h group, half of the MSCs underwent apoptosis, while approximately 80% apoMSCs were observed in the 50 MPa_36 h group. Most cells in the STS group were apoMSCs. **d** The comparison of early apoMSCs (FITC^+ ve^/7-AAD^− ve^) shows the difference between the control group and the 50 MPa_36 h or STS group (*p* < 0.0001, n = 3). The amount of apoMSCs in the 0 MPa_36 h group was higher than that in the control group (*p* < 0.01, n = 3), but lower than that in the 50 MPa_36 h group (*p* < 0.05, n = 3). **e** Immunofluorescence images show the different intensity of living MSCs (stained blue) and apoMSCs (green and red) or dead MSCs (stained red) at 10 × magnification among the groups. At higher magnifications (40×), living MSCs in the control and 0 MPa_36 h group were identified by spindle-shaped flattening, and few apoMSCs were observed. In contrast, many apoMSCs with an intact membrane, PtdSer exposure and even the release of apoptotic bodies were found in the 50 MPa_36 h and STS groups

As shown in the histogram of Fig. 2a, the mean fluorescence intensity (MFI) of Apopxin Green was not only increased by Staurosporine (STS) treatment and 50 MPa_36 h, but also increased in the 0 MPa_36 h group. This could be explained by the sensitivity of MSCs to exposure to rough conditions (closed plastic bag) for a long time. However, the proportion of Apopxin Green-positive cells in the 50 MPa_36 h group was significantly increased in comparison to the 0 MPa_36 h group (*p* < 0.001), while there was no significant difference between the 50 MPa_36 h and STS groups (Fig. 2b). In Fig. 2c, we can visibly observe the dynamic transition of MSCs from a living condition (FITC^− ve^/7-AAD^− ve^) to an apoptotic condition (FITC^+ ve^), even in control groups and most cells are living MSCs (81.9%). Under inappropriate culturing in the 0 MPa_36 h group, a large amount of MSCs became apoptotic; we then observed the reduction of living MSCs there (42.1%). However, after exposure to hydrostatic pressure (50 MPa_36 h group), the proportion of living MSCs was approximately 17.1% and it was considered likely that most cells in the STS group were apoMSCs. Consequently, the comparison of early apoMSCs in Fig. 2d reveals that the proportion of early apoMSCs (FITC^+ ve^/7-AAD^− ve^) in the 50 MPa_36 h group was significantly higher than that in the 0 MPa_36 h group (*p* < 0.05).

Live-imaging of apoptosis/necrosis staining after 3 hours of seeding showed a visibly different population of living MSCs (blue-stained), dead MSCs (red-stained), late apoMSCs (green- and red-stained), and early apoMSCs (green-stained) in all groups (Fig. 2e). Magnified images (10×) revealed similar populations of living cells in the control and 0 MPa_36 h groups. By contrast, most apoptotic cells (green color) were shown in the 50 MPa_36 h and STS groups. Higher magnification images of the control and non-pressurized groups (40×) showed more details of the living MSCs, which were identified by spindle shape flattening. A few apoMSCs or apoptotic bodies can be observed there. In the pressurization or STS groups, we observed the specific morphology of apoMSCs with an intact membrane, PtdSer exposure and even the release of apoptotic bodies. We also found positive signals of blue stained cells in both groups and considered that they were non-viable cells based on a viability assessment (Fig. 5). This phenomenon is related to the remaining intracellular esterase activities and was explained in our previous studies [18, 20].

**Fig. 3:**
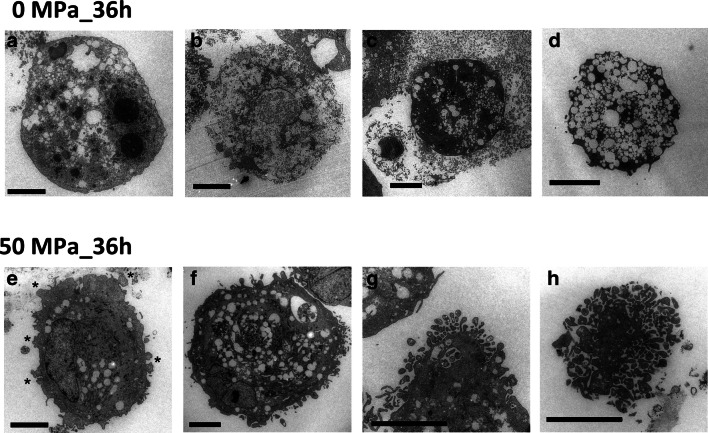
Observation of MSC morphology by transmission electron microscopy (TEM). In the non-pressurization group: **a** living MSCs exhibited a normal morphology with an intact membrane, scant cytoplasm, and round nuclei. **b** Necrotic MSCs with disintegrated nuclei, dilation of organelles, membrane permeabilization and rupture. **c** Apoptotic MSCs with an intact membrane, nuclear fragments, formed of intracellular vacuoles and apoptotic bodies. **d** Autophagic MSCs exhibited an intact membrane, the absence of chromatin condensation, autophagosomes and autolysosome formation in the cytoplasm. In the 50 MPa_36 h pressurization group, only apoMSCs (in different stages of apoptosis) were found. **e** The early phase of apoptosis with reduced cytoplasmic organelles, electron-dense nucleus (pyknosis) and plasma membrane blebbing (asterisks). **f** The intermediate phase shows fragmented nuclei (karyorrhexis) and the formation of intracellular vacuoles with cytoplasmic organelles inside. **g** The final stage shows the release of apoptotic bodies. **h** The hallmark of whole cell fragmentation is shown in the last image. Magnification 6000–12,000×, scale bar: 5 µm

### Tranmission Electron Microscopy (TEM) morphology of apoMSCs

Apoptotic cells show a series of physical changes of plasma membrane blebbing, nucleus defragmentation and cell disintegration into apoptotic bodies that are then engulfed and degraded by phagocytes [21]. Since its ultrastructural morphological characteristics are irrefutable, TEM is considered ‘gold standard’ for proving apoptosis [22]. As shown in Fig. 3, non-pressurized MSCs show distinct responses after 36 h of treatment. In this group we found both viable cells (Fig. 3a) and dying cells, including necrotic cell death (Fig. 3b), apoptotic cell death (Fig. 3c), and autophagic cell death (Fig. 3d). Interestingly, in the 50 MPa_36 h pressurization group, only apoMSCs in different stages of apoptosis were found. Figure 3e shows the early phase of apoptosis with an electron-dense nuclei (pyknosis) and many blebs on the cell surface (indicate by asterisks). In the intermediate phase (Fig. 3f), cell nuclei are fragmented (karyorrhexis) and intracellular vacuoles containing cytoplasmic organelles are formed. Then, in the final stage, apoptotic bodies are released (Fig. 3g), and the hallmark of cell fragmentation is observed (Fig. 3h).

### Assessment of caspase activity and nuclear defragmentation in apoMSCs

**Fig. 4:**
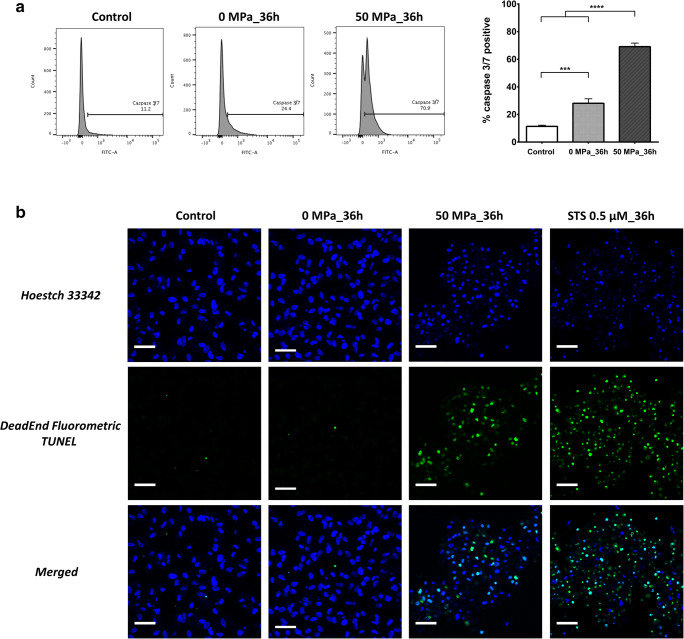
Nuclear defragmentation and activated caspase-3/7 are involved in HHP treatment.** a** A representative flow cytometry histogram shows a higher percentage of activated caspase-3/7-positive MSCs in the 0 MPa_36 h group in comparison to the control group (28.1% vs. 11.4%, *p* < 0.001, n = 3). After 36 h of pressurization at 50 MPa, the amount of activated caspase-3/7 detected in MSCs had clearly increased in comparison to the non-pressure group (69.1%, *p* < 0.0001, n = 3). **b** DeadEnd Fluorometric TUNEL staining showed that a large number of MSCs in the 50 MPa_36 h and STS treatment groups had DNA fragmentation, while few MSCs showed fragmentation in the control or 0 MPa_36 h groups. In addition, the number of oval-shaped, intact nuclei that were counterstained by Hoestch 33,342 revealed that most MSCs in the control and 0 MPa_36 h group were alive. In contrast, the changes in the nuclear morphology of the MSCs in the 50 MPa_36 h and STS groups suggest that they were dying cells

Caspases play a critical role in protein cleavage in the apoptotic pathway, in which caspase-3 and caspase-7 are considered the most important executioner caspases, which are activated by any of the initiator caspases. The catalytic activity of executioner caspases is responsible for many of the morphological and biochemical correlates of apoptosis, including DNA fragmentation, PtdSer exposure, and the formation of apoptotic bodies [23]. In this study, MSCs in closed bags (0 MPa_36 h) showed a higher percentage of activated caspase-3/7 (Fig. 4a) in comparison to cells cultured in an incubator in the control group (28.1% vs. 11.4%, *p* < 0.001, n = 3). After treatment at 50 MPa for 36 h, the amount of activated caspase-3/7 in MSCs showed a marked increase (69.1%, *p* < 0.0001). This result revealed the effective impact of HHP treatment for inducing MSCs undergo to apoptosis in comparison to the non-pressurized group or control group.

Another hallmark of apoptosis is internucleosomal cleavage of genomic DNA into small fragments, which were then detected by a TdT-mediated dUTP-biotin nick end labeling (TUNEL) assay. TUNEL staining utilizes the ability of the enzyme terminal deoxynucleotidyl transferase (TdT) to incorporate labeled dUTP onto the free 3’-hydroxyl termini of fragmented genomic DNA [24]. As shown in Fig. 4b, DeadEnd Fluorometric TUNEL-stained images demonstrated DNA fragmentation in many of the MSCs in the 50 MPa_36 h and STS treatment groups; in contrast, few MSCs in the control and 0 MPa_36 h groups showed DNA fragmentation. Furthermore, the presence of oval-shaped, nuclei with an intact membrane, which were stained by Hoestch 33,342, showed that most of the cells in the control and 0 MPa_36 h groups were alive. In contrast, the changes in the nuclear morphology of the MSCs in the 50 MPa_36 h and STS groups suggested that were dying cells.

### Viability assessments of pressurized MSCs

**Fig. 5:**
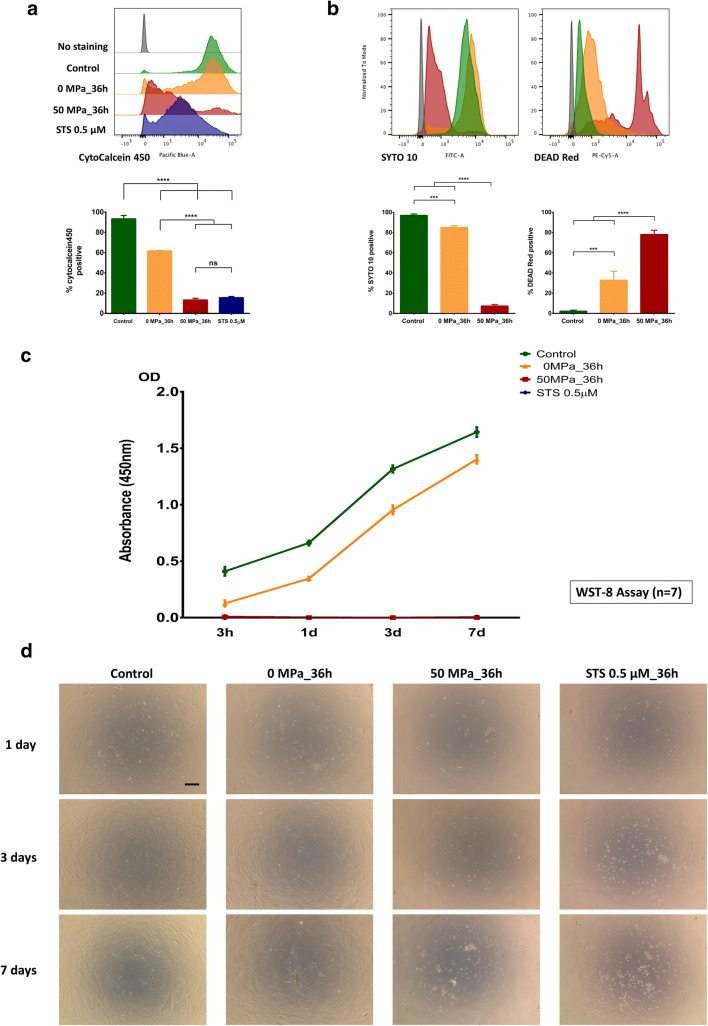
Viability assessment of MSCs after pressurization.** a** A representative CytoCalcein 450 histogram shows that there was no significant difference in cell survival between MSCs in the 0 MPa_36 h and control groups; this level was markedly different from the 50 MPa_36 h or STS groups. Accordingly, the ratios of CytoCalcein 450-positive cells in the in 50 MPa_36 h and STS treatment groups were actually lower in comparison to the 0 MP_36 h (13.3% vs. 61.7%, *p* < 0.0001, n = 3) and the control group (13.3% vs. 93.4%, *p* < 0.0001, n = 3). **b** The SYTO 10 fluorescence histogram and comparison of the positive ratio revealed the similarity of MSC nuclei staining in the control and 0 MPa_36 h groups, while a profound difference was observed in the 50 MPa_36 h group (*p* < 0.0001, n = 3). In contrast, the results of DEAD Red staining indicated that approximately 33% of MSCs in the 0 MPa_36 h group were dead cells; in contrast, approximately 78% of the MSCs in the 50 MPa_36 h group were dead cells (*p* < 0.0001, n = 3). **c** The measurement of absorbance at 450 nm in the WST-8 assay shows that the MSCs in the 0 MPa_36 h group were markedly weaker than those in the control group at 3 h after culturing; however, they subsequently proliferated alongside normal MSCs in the control group until day 7. Nonetheless, neither the signal of viable MSCs in the 50 MPa_36 h or STS group was detected after up to 7 days of cells seeding. **d** Live-imaging microscopy revealed no living cells in the 50 MPa_36 h and STS groups at any of the time points, while numerous MSCs in the 0 MPa_36 h group were viable, their original characteristics remained and they showed strong proliferation, similarly to normal MSCs (control group). Magnification 4×, scale bar 250 µm

We used CytoCalcein 450 staining in an Apoptosis/Necrosis assay (#ab176749, Abcam) and Live/Dead staining (L-7013, Molecular Probes) to investigate the viability of MSCs after pressurization. As shown in Fig. 5a, the CytoCalcein 450 histogram revealed that there was no significant difference in cell survival of MSCs in the 0 MPa_36 h and control groups, while there was a marked difference in survival in the 50 MPa_36 h and STS treatment groups. Consequently, the ratio of CytoCalcein 450-positive MSCs in the 50 MPa_36 h and STS treatment groups was actually lower in comparison to that in the 0 MP_36 h (13.3% vs. 61.7%, *p* < 0.0001, n = 3) and control (13.3% vs. 93.4%, *p* < 0.0001, n = 3) groups. Moreover, in a Live/Dead staining assay, SYTO 10, a green fluorescent nucleic acid stain, is a highly membrane-permeant dye that labels all cells, including those with intact plasma membranes. As shown in Fig. 5b, which demonstrates the expression of SYTO 10, we the staining of MSC nuclei in control and 0 MPa_36 h groups was similar, while that in in the 50 MPa_36 h group was profoundly different. This could be explained by the occurrence of nuclear fragmentation in the apoMSCs of the pressurized group. In contrast, DEAD Red is a cell-impermeant fluorescent nucleic acid stain that only labels cells with compromised membranes (referred to as dead cells). In Fig. 5b, approximately 33% of MSCs in the 0 MPa_36 h group were dead in comparison to approximately 78% of MSCs in the 50 MPa_36 h group (*p* < 0.0001, n = 3).

In addition, an WST-8 assay and inverted microscopy were used to confirm completely irreversible MSC death and proliferation. As shown in Fig. 5c, we found that the MSCs in the 0 MPa_36 h group were markedly weaker than those in the control group at 3 h after culturing, but subsequently proliferated alongside normal MSCs in control group until day 7. Nonetheless, neither signal of viable MSCs was detected at up to 7 days of cell seeding in the 50 MPa_36 h and STS treatment groups. Besides, live-imaging microscopic observation of pressurized MSCs (Fig. 5d) detected no living cells in the 50 MPa_36 h and STS groups at any of the time points, while most MSCs in the 0 MPa_36 h group were viable, with their original characteristics, and showed good proliferation over time, similarly to normal MSCs (control group). As a consequence, we considered that persistent HHP treatment at 50 MPa truly induced MSC death.

## Discussion

Numerous studies have reported that apoptosis usually occurs after HHP treatment at approximately 100 MPa. This phenomenon has been observed in many cell lines, including murine erythroleukemia (MEL) cells, human lymphoblasts, B35, PC12 and retinal ganglion cell lines [15]. The molecular biology of high pressure-induced apoptosis was demonstrated through the activation of caspase-3, via both extrinsic and intrinsic pathways [25]. In the current study, we confirmed the appearance of cleaved caspase-3/7, executioner caspases are considered irreversible and play a critical role in all of the morphological changes of apoptotic cells, including DNA fragmentation and PtdSer membrane exposure [23]. As shown in Fig. 4a, besides the clear elevation of activated caspase-3/7 of apoMSCs in the 50 MPa_36 h group, the elevation was also observed in 0 MPa_36 h group. Furthermore, increased PtdSer exposure, which was detected by Apopxin Green, could also be observed in the 0 MPa_36 h group (Fig. 2). These results indicated that the suspended culture condition within a closed bag for a long period of time contributed to the stimulation of programmed cell death of MSCs. Possible causes might be any non-optimal culture condition, or combination thereof, including nutrient starvation, pH changes, hypoxia, or cell aggregation [26]. Accordingly, necrosis, apoptosis or autophagy of MSCs in the 0 MPa_36 h group could be spontaneously triggered. The ultrastructure morphology of MSCs in these states is shown in Fig. 2b and d. Does this condition impact the effect of persistent HHP treatment? Notably, we did not find any of the above MSC death phenomena in the 50 MPa_36 h group, with the exception of apoptotic bodies and apopMSCs of different morphological stages (Fig. 2e and h). It is not clear whether the impact of hydrostatic pressure alone or the concomitant impact of all factors inside are responsible for this effect and further studies are needed.

As shown in Fig. 4b, TUNEL staining clearly demonstrated the difference of MSCs with/without pressurization after 36 hours of treatment. Indeed, we detected diverse TUNEL signals (FITC-positive) in the apoMSCs in the 50 MPa group, similarly to the STS treatment group. Although TUNEL staining is very sensitive due to the ability to detect a single cell via fluorescence microscopy, it is important to recognize that this staining is not limited to the detection of apoptotic cells [27]. Because TUNEL staining is a non-specific label all free 3’-hydroxyl termini, it will also detect non-apoptotic cells, including cells undergoing necrotic degeneration, cells undergoing DNA repair, cells damaged by mechanical forces, and even cells undergoing active gene transcription. Thus, this should be generally considered a method for detecting DNA damage (DNA fragmentation or others), that can be applied more specifically when used in conjunction with secondary apoptosis-specific assays [28].

Apoptosis has long been considered a form of ‘silent’ cell death, in contrast to necrosis, which frequently induces inflammation via the release of danger-associated molecular patterns (DAMPs). However, this notion has changed, and apoptosis has gradually been shown to participate in communication with neighboring cells to contribute to survival or apoptosis and remodeling of the surrounding tissues [29]. Moreover, Thum et al. proposed that the efferocytosis of apoMSCs may cause the downregulation of the innate and adaptive immunity in the local immune response [30]. Accordingly, various biotechnology and cell therapy industries are currently attempting to develop this subcellular therapeutic machinery (in a naïve or modified state) for regenerative medicine, as substitutes for intact cell therapy, and as intelligent targeted drug delivery carriers [11]. In this study, our data show that HHP treatment is a convenient process that can effectively induce apoptosis in MSCs. Furthermore, this process is considered to be as no chemical reagents are used; thus it would be simple to apply in manufacturing of therapeutic products [14]. Nevertheless, further studies should be performed to investigate the characteristics of extracellular vesicles derived from HHP induced-apoMSCs and their potential application in clinical research.

## Data Availability

All data generated or analysed during this study are included in this published article.

## Abbreviations

Apoptotic MSCs: apoMSCs
CAFs: carcinoma associated fibroblasts
DAMPs: danger-associated molecular patterns
EVs: extracellular vesicles
HHP: high hydrostatic pressure
MSCs: mesenchymal stem cells
MSC-EVs: MSC-derived extracellular vesicles
PtdSer: phosphotidylserine
STS: Staurosporine
TEM: Transmission electron microscopy
TUNEL: terminal deoxynucleotidyl transferase dUTP nick-end labeling
